# Functional analysis of the BEige and Chediak-Higashi domain gene Mp*SPIRRIG* in *Marchantia polymorpha*

**DOI:** 10.3389/fpls.2022.915268

**Published:** 2022-09-23

**Authors:** Eva Koebke, Lisa Stephan, Markus G. Stetter, Martin Hülskamp

**Affiliations:** Botanical Institute, University of Cologne, Cologne, Germany

**Keywords:** *SPIRRIG*, *Marchantia polymorpha*, BEACH domain protein, salt response, membrane trafficking

## Abstract

BEige and Chediak–Higashi domain containing proteins (BDCPs) have been described to function in membrane-dependent processes in eukaryotes. This role was also observed for the BDCP SPIRRIG (SPI) in the model plant *Arabidopsis thaliana* in the context of cell morphogenesis. Additionally, *AtSPI* was found to control salt stress resistance by mediating mRNA stability and salt stress-dependent processing body formation. In this work, we utilize an evolutionarily comparative approach to unravel conserved, basal BDCP functions in the liverwort *Marchantia polymorpha*. Our phenotypic and physiological analyses show that Mp*SPI* is involved in cell morphogenesis and salt resistance regulation, indicating that both functions are evolutionarily conserved between the two species. Co-localization was found with endosomal and P-body markers, suggesting links to membrane-dependent processes and mRNA metabolism. Finally, we present transcriptomics data showing that *AtSPI* and Mp*SPI* regulate orthologous genes in *A. thaliana* and *M. polymorpha*.

## Introduction

Beige and Chediak-Higashi (BEACH) domain containing proteins (BDCPs) represent a conserved gene family in eukaryotes. BDCPs function as scaffolding proteins in membrane fission and fusion events, including vesicle transport, receptor signaling, apoptosis, and autophagy (Cullinane et al., 2013). Genetic and molecular studies in *A. thaliana* revealed a role in vacuolar and Endosomal Sorting Complex Required for Transport (ESCRT)-mediated membrane trafficking of the BDCP gene *AtSPIRRIG* (*AtSPI*; Saedler et al., 2009; Steffens et al., 2017). AtSPI exhibits the classical BDCP domain structure with the characteristic and highly conserved C-terminal end, comprising a pleckstrin homology (PH) domain to bind phospholipids (Cullinane et al., 2013); the BEACH domain, which potentially serves as a ligand-binding site by interacting with the PH domain (Jogl et al., 2002); and WD40 repeats to mediate protein–protein interactions (Stirnimann et al., 2010).

*Atspi* mutants were initially isolated in an EMS mutagenesis screen due to their weak, distorted trichome phenotype (Hülskamp et al., 1994). Further phenotypic characterization of the mutants revealed *Atspi* to exhibit trichomes with shorter branches and reduced stalk length, less complex epidermal pavement cells, and shorter root hairs and hypocotyls (Saedler et al., 2009). AtSPI has a function in maintaining vacuolar integrity (Saedler et al., 2009) and endosomal transport *via* ESCRT-mediated membrane trafficking through direct interaction with components of the machinery (Steffens et al., 2017). In addition, a role of AtSPI in salt stress-dependent mRNA regulation was uncovered (Steffens et al., 2015): AtSPI interacts with the processing body (P-body) component DECAPPING PROTEIN 1 (AtDCP1) and localizes to and promotes the assembly of P-bodies under salt stress conditions (Steffens et al., 2015). Furthermore, AtSPI was demonstrated to be involved in stabilizing and recruiting salt stress-dependent mRNAs to P-bodies (Steffens et al., 2015). Finally, the finding that mutations in *AtSPI* lead to salt hypersensitivity proved a biologically relevant function of AtSPI in the *A. thaliana* salt stress response (Steffens et al., 2015).

Evolutionary comparison of SPI between *A. thaliana* and *Arabis alpina* demonstrated that the role of SPI in cell morphogenesis and endosomal pathways is conserved between these two Brassicaceae species (Stephan et al., 2021). This is evident from SPI co-localization and interaction with ESCRT components and a similar range of phenotypes. Moreover, a link to P-bodies is suggested by direct protein–protein interaction with P-body markers (Stephan et al., 2021).

In this study, we characterize a *spirrig* T-DNA mutant in the liverwort *Marchantia polymorpha* (Mp*spi*), isolated by Honkanen et al. (2016). During the last decade, *M. polymorpha* has been established as a new molecular model organism to assess comparisons over large evolutionary distances (Mishler and Churchill, 1984; Shimamura, 2016). It enables researchers to study the evolution of land plants, and concomitantly, learn about the basal, minimal genetics coming along with land colonization (Bowman et al., 2007; Ishizaki et al., 2016). The relatively small, fully sequenced genome with a low level of redundancy (Bowman et al., 2017), combined with the dominant, haploid gametophytic life phase of *M. polymorpha*, offers optimal genetic and morphological conditions for reverse and forward genetics. Moreover, *M. polymorpha* can be transformed using Agrobacterium (Ishizaki et al., 2008; Kubota et al., 2013), and it is accessible for genome editing *via* homologous recombination (Ishizaki et al., 2013) and CRISPR/Cas9 (Sugano et al., 2014, 2018). Finally, quick and easy tools to analyze the localization, interactions, and functions of proteins of interest, as well as cell morphology, using transient biolistic transformation and staining methods, are available (Westermann et al., 2020).

Here, we present an evolutionarily comparative functional study of Mp*SPI*. We show that mutant plants are hypersensitive to salt stress, indicating that the dual role of At*SPI* in cell morphogenesis and salt stress response is evolutionarily conserved. In addition, using a comparative RNAseq approach, we present a common set of homologous genes regulated by *A. thaliana* and *M. polymorpha* SPI, strongly suggesting functional conservation of BDCP function between the two species.

## Materials and methods

### Plant materials, growth conditions, and stress treatments

The T-DNA insertion in the Mp*spirrig* mutant (corresponding designations: Mp*spi-2* and ST17-11; Honkanen et al., 2016) was confirmed by sequencing the amplicon generated with the gene-specific primer 5′-CGAGCCGACTTACCCCTAAT-3′ and the T-DNA (pCAMBIA 1300) left border primer 5′-CAGATAAGGGAATTAGGGTTCCTATAGG-3′. The male sex of the mutant line was confirmed with the male-specific primer pair 5′-CCAAGTGCGGGCAGAATCAAGT-3′ and 5′-TTCATCGCCCGCTATCACCTTC-3′, amplifying *rbm27* (Fujisawa et al., 2001).

Plants of the mutant line, the corresponding male ecotype Tak-1, the female ecotype Tak-2, and F1 crossings of Tak-1 and Tak-2 (Tak-1 × Tak-2) were propagated vegetatively under axenic conditions by growing gemmae on Johnson’s medium (Johnson et al., 1957) supplemented with 0.8% plant agar under long-day conditions (16 h light/8 h darkness cycle) and white light (60 μmol m^−1^ s^−1^) at 21 ± 2°C.

In order to assess the rhizoid growth, gemmae were grown vertically on solid Johnson’s medium without supplements or supplemented with 50 mM NaCl under normal conditions for 7 days.

### Cloning and plasmids

Total RNA was extracted from 14-day-old Tak-1 thalli using TRI reagent (Ambion Life Technologies). RNA was treated with DNaseI (Thermo Scientific) and subjected to oligo(dT)_20_ cDNA synthesis using the SuperScript™ III First-Strand cDNA Synthesis Kit (Thermo Scientific). Coding sequences of MpSPI PBW (Mp2g15800), MpSPI PBWF (Mp2g15800), MpLIP5 (Mp1g06880), and MpSKD1 (Mp8g01610) were amplified from Tak-1 cDNA (Primer list, Supplementary Table S6). MpDCP2 (Mp8g16420), MpRAB5, and MpARA6 plasmids have been described before (Westermann et al., 2020). mCH-AtUBP1B was kindly provided by A. Steffens. AtKRP1-CFP was kindly provided by M. Jakoby. Coding sequences were cloned into Gateway vectors pDONR201 and pDONR207 (Invitrogen) and subsequently transferred to respective expression vectors: for localization studies, we used pENSG-YFP/CFP, pEXSG-YFP/CFP (Feys et al., 2005), pAMARENA/pAUBERGINE (M. Jakoby, GenBank ID: FR696418) and pMpGWB406 (Ishizaki et al., 2015); yeast two-hybrid assays were performed using pAS/pACT (Clontech); and Bimolecular Fluorescence Complementation (BiFC) was performed using pSYN/pSYC (Jakoby et al., 2006) and pCL112/113 (provided by J. F. Uhrig).

### Protein–protein interaction assays

The protocols for yeast two-hybrid assays were described before (Gietz et al., 1995). Positive interactions were selected on plates containing dropout interaction media lacking leucine, tryptophan, and histidine, supplemented with increasing concentrations of 3-Amino-1,2,3-Triazole (3-AT) up to 50 mM. BiFC assays were performed as described previously (Westermann et al., 2020).

### Plant transformation

Transient biolistic transformation of *M. polymorpha* thalli was performed as described before (Westermann et al., 2020). Stable transformation of regenerating *M. polymorpha* thalli fragments with *Agrobacterium tumefaciens* (GV3101 pMP90RK) was conducted as described by Kubota et al. (2013).

### Staining procedures

Fluorescein diacetate (FDA) stainings of young gemmae (5 days old) were performed as described before (Westermann et al., 2020).

### Microscopic analysis

Microscopic observation was carried out with a Leica MZ 16F fluorescence binocular or a Leica TCS SP8 confocal laser scanning microscope using an HC PL APO 20 ×/0.75 IMM CORR CS2 objective. The excitation and emission of the different fluorophores were performed as described before (Westermann et al., 2020).

### Transcript analysis

Total RNA was extracted from three biological replicates of 14-day-old Tak-1 and Mp*spirrig* thalli grown under normal conditions using TRI reagent (Ambion Life Technologies). RNA integrity was confirmed on a bleach gel (Aranda et al., 2012). 1 μg of DNaseI-treated RNA was subjected to oligo(dT)_20_ cDNA synthesis using the SuperScript^™^ III First-Strand cDNA Synthesis Kit (Thermo Scientific). Quantitative Real-Time PCR (RT-qPCR) was performed in a QuantStudio 5 System (ABI/Life Technologies) using plates (96 well, 0.2 ml) and cover foil (Opti-Seal Optical Disposable Adhesive, BIOplastics) and SYBR Green reagent (Thermo Fisher Scientific). Subsequent analysis was conducted with the QuantStudio TM Design and Analysis Software version 1.4.1 (ABI/Life Technologies) and Excel 2016. The average of three biological and three technical replicates was calculated. Primer efficiencies were determined using a cDNA dilution series of 1:10, 1:20, 1:40, 1:80, 1:160, and 1:320. Reference gene primers Mp*ADENINE PHOSPHORIBOSYL TRANSFERASE 3* (Mp*APT3*) and Mp*ACTIN7* (Mp*ACT7*) were described before (Saint-Marcoux et al., 2015). The efficiency of the primer pair for Mp*SPIRRIG* (Primer list, supplemental) was accepted with an efficiency of 80–120% and a correlation between −1 and − 0.99. Normalization against two reference genes was performed according to the geNorm manual (Vandesompele et al., 2002).

### RNA sequencing and transcriptome analysis

Total RNA for RNAseq analysis was extracted from three biological replicates of 14-day-old Tak-1 and Mp*spirrig* thalli grown under control conditions using TRI reagent (Ambion Life Technologies). Two μg of DNaseI-treated, quality-controlled RNA (RIN > 7, OD260/280 = 1.8–2.1, OD260/230 > 1.5) were sent to the Cologne Center for Genomics (CCG) for paired-end 100 bp short-read sequencing. Raw reads were filtered and trimmed using trimmomatic (v 0.39) with SLIDINGWINDOW:4:15 MINLEN:35 LEADING:5 TRAILING:5 (Bolger et al., 2014), and data quality was assessed using MultiQC (v 1.7; Ewels et al., 2016). Filtered reads were aligned to the MpTak1_v5.1 reference genome (Montgomery et al., 2020) using STAR (v 2.7.3a; Dobin et al., 2013), and reads per gene were counted using the htseq-count function of HTSeq (v 0.11.3; Anders et al., 2015). We further used DESeq2 (v 1.24.0; Love et al., 2014) to compare read counts between Tak-1 and the Mp*spirrig* mutant. We filtered genes for a minimum of ten reads in at least three of the six samples. Differentially expressed genes (DEGs) were filtered using the adjusted value of p cutoff of 0.01 and a log_2_ fold change of 1. To test the overlap between DEGs in the Mp*spirrig* mutant and DEGs in *Atspirrig,* we used PhytoMine (v.13)^1^ through the intermine (v 1.11.0) Python API to identify homologs between *M. polymorpha* and *A. thaliana* and applied a hypergeometric test for overlap in R. Sequence reads are available on the European Nucleotide Archive under project number PRJEB51622. *M. polymorpha* Gene Ontology (GO) term enrichment analysis was performed as previously described (Busch et al., 2019) with agriGO v.2 (standard settings; http://systemsbiology.cau.edu.cn/agriGOv2/; Tian et al., 2017) using gene IDs for the MpTak1_v3 reference genome.

## Results

### The *Marchantia polymorpha* Mp*spi* mutant

The BEACH domain proteins in eukaryotes cluster into four groups (A–D; Saedler et al., 2009) with similar domain structures (Supplementary Figure S1). *AtSPI* belongs to group A and is well conserved throughout the plant kingdom (Supplementary Figure S1; Stephan et al., 2021). Group A members share a Laminin G/Concanavalin A superfamily domain but lack additional domains found in the other groups. BLAST analysis (marchantia.info, MpTak1v5.1) revealed a total of five BDCPs in *M. polymorpha,* and phylogenetic tree construction indicates that Mp*SPI* is the closest homolog to *AtSPI* (Supplementary Figure S1).

**Figure 1:**
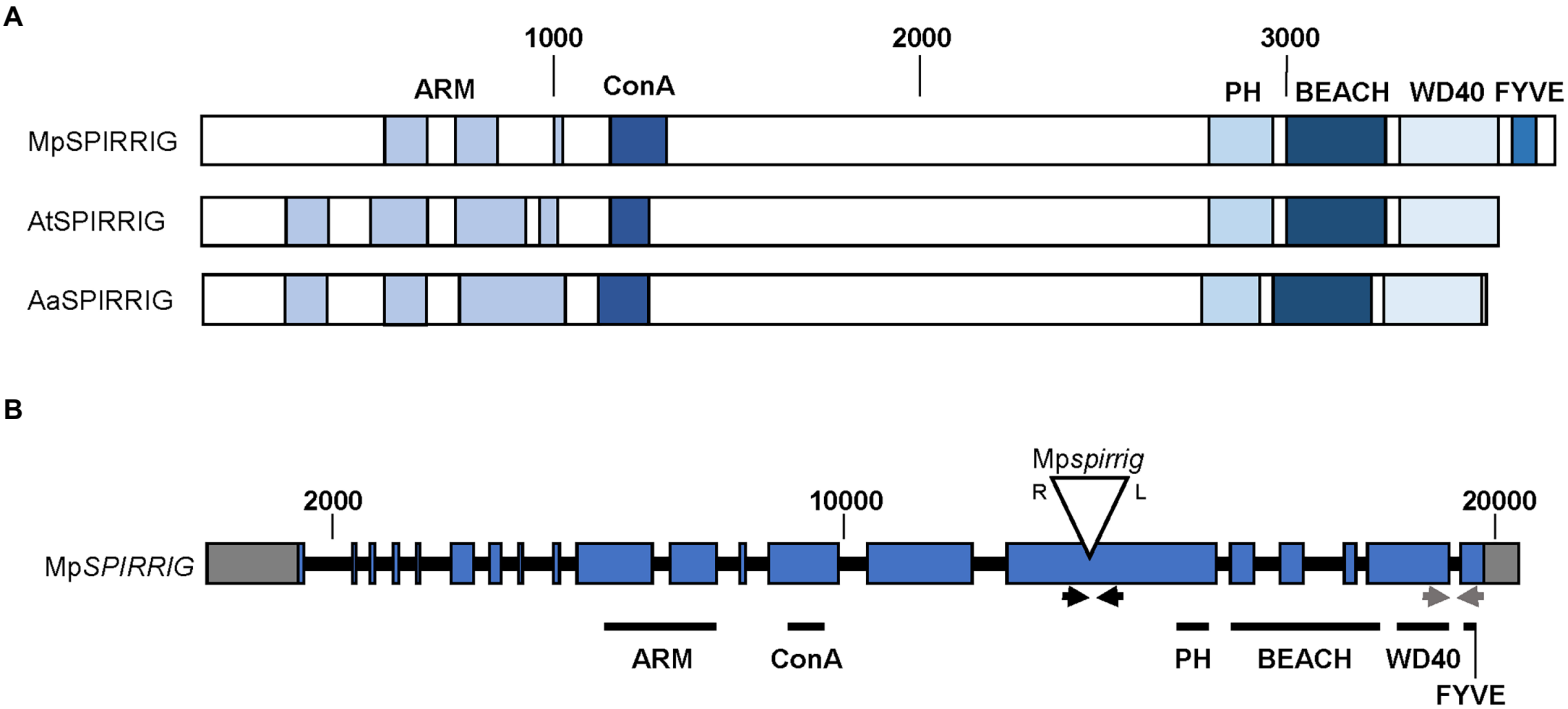
Gene and protein structure of Mp*SPIRRIG*. **(A)** Comparison of the Mp*SPIRRIG*, AtSPIRRIG, and AaSPIRRIG protein structures. Sizes are indicated by amino acid (aa) numbers. The three homologs are similar in length: MpSPI (3,766 aa), AtSPI (3,601 aa), AaSPI (3,570 aa). ARM = Armadillo repeats, ConA = Concanavalin A-like lectin domain, PH = pleckstrin homology domain, BEACH = BEige and Chediak-Higashi domain, WD40 = WD40 repeats, FYVE = FYVE domain. **(B)** Gene structure of Mp*SPI* including the T-DNA insertion site in Mp*spi*. Mp*SPI* has 20 exons (indicated in blue). The T-DNA insertion is indicated by the triangle, R and L mark the T-DNA borders. Gray boxes show UTRs and black lines depict introns. The arrows indicate primer pairs used for RT-PCR (black) and qPCR (gray).

The overall structure of the Mp*SPI* gene is similar to the previously investigated plant *SPI* homologs of *A. thaliana* and *A. alpina*; however, Mp*SPI* has an additional FYVE domain downstream of the PBW domain (Figure 1A). FYVE domains are known to target membranes by binding to phosphatidylinositol 3 phosphate (PtdIns(3)P) and are functionally connected to vacuolar protein sorting and endosome function (Gaullier et al., 1998).

The male T-DNA mutant line Mp*spi*, isolated by Honkanen et al. (2016), harbors a single T-DNA insertion in the 15th exon of Mp*SPI* (Honkanen et al., 2016; Figure 1B) upstream of the region coding for the characteristic C-terminal PBW domain. RT-qPCR experiments using primers located downstream of the insertion site revealed no significant differences between wild type and mutants (Supplementary Figure S2A), indicating that this insertion does not affect the expression levels of Mp*SPI*. The produced RNA, however, is incomplete. This is evident from qualitative RT-PCR data, showing that a primer pair spanning the T-DNA insertion site generates no bands in Mp*spi* (Supplementary Figure S2B).

### Morphological phenotypes of Mp*spirrig* gemmae

**Figure 2:**
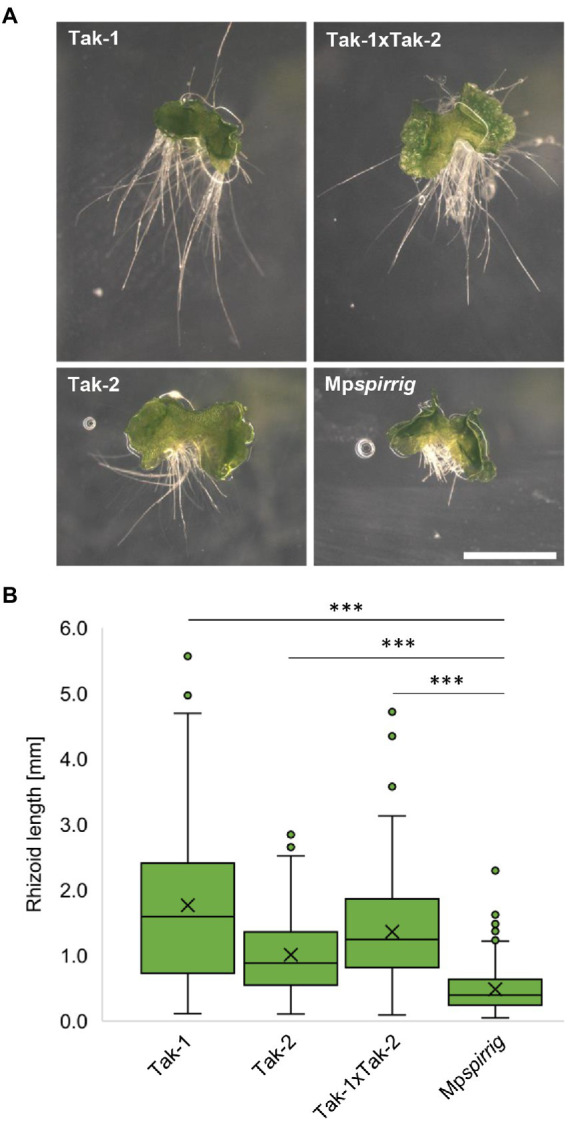
Mp*spi* has a severe short-rhizoid phenotype. **(A)** Representative images of gemmalings grown vertically on Johnson‘s medium for 7 days. Scale bar: 2 mm. **(B)** Rhizoid length of seven-day-old gemmalings. *N* ≥ 240. Significance was tested by ANOVA at *p* ≤ 0.001 (^***^).

Honkanen et al. (2016) initially identified Mp*spi* by its short rhizoid phenotype. We confirmed this phenotype in seven-day-old gemmalings. Mp*spi* showed a clear reduction in rhizoid length compared to Tak-1 (3.6 fold), Tak-2 (2.1 fold), and Tak-1xTak-2 F1 gemmalings (2.8 fold; Figure 2A). Statistical analysis revealed that this reduction is significant (*p* ≤ 0.001; Figure 2B).

As *Atspi* mutants in Arabidopsis display fragmented vacuoles (Saedler et al., 2009), we stained five-day-old gemmae with fluorescein diacetate (FDA). Vacuoles in rhizoids were indistinguishable between Tak-1, Tak-2, Tak1 × Tak-2 crossings, and Mp*spi* (Supplementary Figure S3), similar as found for *A. alpina spirrig* mutants (Stephan et al., 2021).

### Salt stress phenotype of the Mp*spirrig* mutant

**Figure 3:**
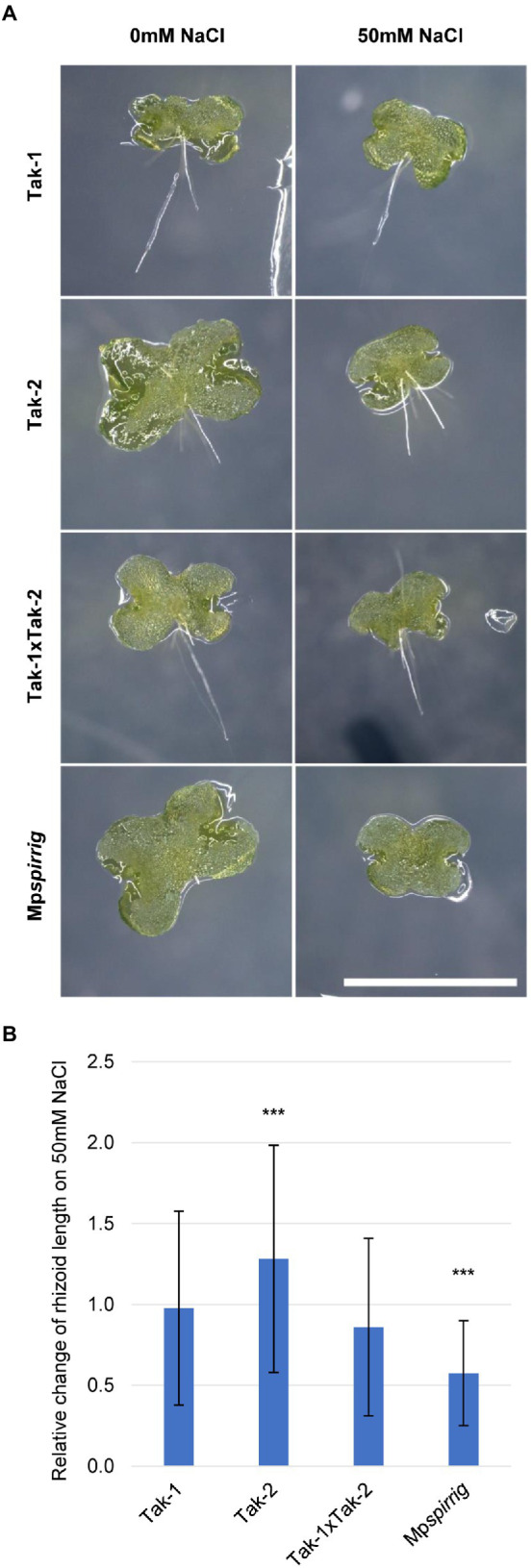
Mp*spirrig* is salt hypersensitive. **(A)** Rhizoid growth is strongly impaired in 4-day-old gemmalings of Mp*spirrig* compared to Tak-1, Tak-2, and Tak1xTak-2. Scale bar: 2 mm. **(B)** Comparison of rhizoid growth under salt stress (*n* ≥ 160) and control conditions (*n* ≥ 240) on 7-day-old gemmalings. Significance was tested by ANOVA at *p* ≤ 0.001 (***).

*A. thaliana* and *A. alpina spirrig* mutants show a hypersensitive response to salt stress. In order to investigate whether Mp*SPI* is involved in the *M. polymorpha* salt stress response, we examined the effect of 50 mM NaCl on the rhizoid length of Mp*spirrig*. Rhizoid length was analyzed after 7 days. We found a significant reduction of the rhizoid length under salt stress conditions (Figure 3). This indicates that Mp*spi* is salt hypersensitive and that Mp*SPI* is relevant for the salt stress response in *M. polymorpha*.

### MpSPI interacts with ESCRT components

The association of *A. thaliana* and *A. alpina* SPI with membrane-dependent processes is suggested by co-localization and direct protein–protein interactions with ESCRT components (Steffens et al., 2017; Stephan et al., 2021). To test whether SPI in *M. polymorpha* shares this behavior, we first performed co-localization assays in *M. polymorpha* epidermal cells. Toward this end, we focused on MpSPI, MpLIP5, and MpSKD1, for which co-localization and interactions were reported for *A. thaliana* and *A. alpina* (Steffens et al., 2017; Stephan et al., 2021).

**Figure 4:**
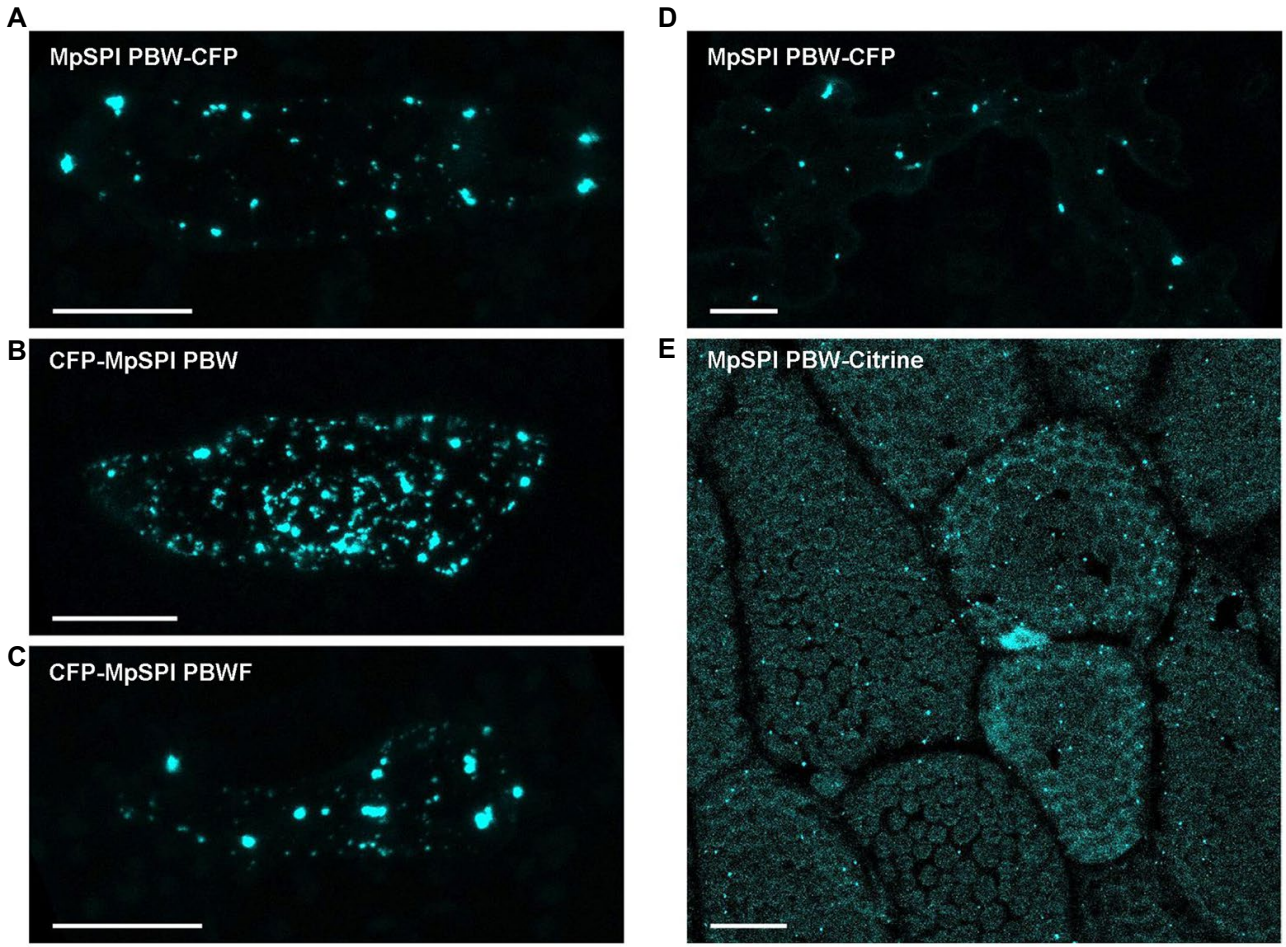
MpSPI PBW localizes to dot-like structures under non-stress conditions. **(A)** Transiently expressed, CFP tagged MpSPI PBW localizes to punctate structures in Tak-1 epidermal cells under normal conditions **(B)** irrespective of the position of the fluorescent tag and **(C)** the addition of the FYVE domain. Scale bars: 25 μm. **(D)** Transiently expressed, CFP tagged MpSPI PBW localizes to punctate structures in *A. thaliana* Col-0 epidermal cells under normal conditions. Scale bar: 25 μm. **(E)** Citrine tagged MpSPI PBW was stably transformed into regenerating Tak-1 thalli. Image shows a 5-day-old gemmae derived from the stable line. Scale bar: 25 μm.

Co-localization experiments were done with protein fragments of MpSPI as we were not able to construct the about 12 kb long full-length CDS. The fragments were chosen to fit Arabidopsis and Arabis fragments used in previous studies to facilitate comparison. MpSPI PBW alone localizes to distinct cytoplasmic dots in transient assays as well as in stably transformed Tak-1 thalli (Figures 4A,E). The localization was neither dependent on the position of the fluorescent tag nor on the presence of the C-terminal FYVE domain (Figures 4B,C). This localization behavior differs from AtSPI to AaSPI, which are evenly distributed in the cytoplasm (Steffens et al., 2017; Stephan et al., 2021). To test whether this is a property of MpSPI or the cellular environment, we studied MpSPI localization in transiently transformed *A. thaliana* epidermal cells. Here as well, MpSPI was localized to dots indicating that the localization behavior is not primarily triggered by the cellular environment (Figure 4D).

**Figure 5:**
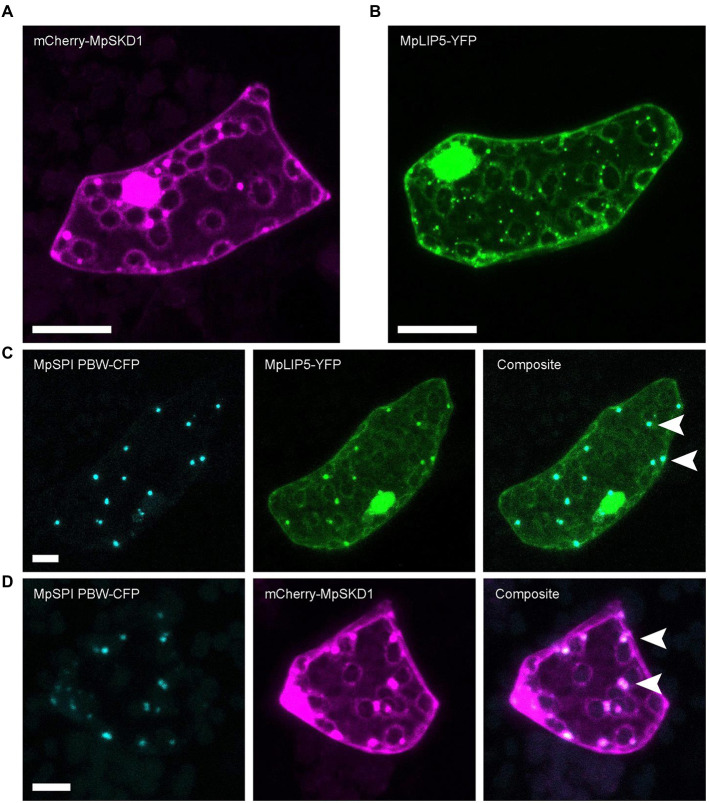
MpSPI PBW co-localizes with ESCRT components in *M. polymorpha.* **(A)** Dot-like localization of transiently expressed mCherry-MpSKD1 in Tak-1 epidermal cells. **(B)** Dot-like localization of transiently expressed MPLIP5-YFP in Tak-1 epidermal cells. **(C)** Transient co-expression of MpSPI PBW and MpLIP5-YFP in Tak-1 epidermal cells. **(D)** Transient co-expression of MpSPI PBW and MpSKD1 in Tak-1 epidermal cells. Arrowheads indicate areas of co-localization. Scale bars = 20 μm.

MpLIP5 and MpSKD1 both localize to cytoplasmic, dot-like structures (Figures 5A,B), which co-localize entirely with MpSPI PBW in double transformations (Figures 5C,D). In contrast, co-localization experiments with the endosomal marker proteins MpRas-related in brain 5 (MpRAB5) and MpARA6 (also known as RABF1) revealed only partial overlap supporting a direct interaction of MpSPI with MpLIP5 and MpSKD1, rather than a general localization to endosomal structures (Supplementary Figure S4, S5).

To test this, we assessed direct protein–protein interactions of MpSPI with ESCRT components in pairwise yeast two-hybrid assays. While no interaction between MpSPI PBW/PBWF and MpSKD1 was detectable, we found a strong interaction between MpSPI PBW/PBWF and MpLIP5 (Supplementary Table S1). However, this interaction could not be independently confirmed. Bimolecular fluorescence complementation (BiFC) assays in cells of *M. polymorpha* (Supplementary Figure S6), *A. thaliana*, *A. porrum*, and *N. benthamiana* showed no interactions of MpSPI PBW with MpSKD1 or MpLIP5. Moreover, Förster Resonance Energy Transfer-Acceptor Photobleaching (FRET-AP) and pull-down assays experimentally did not succeed in our hands. Expressed MpLIP5 was non-specifically bound by any protein tag, and it was not possible to detect MpSPI PBW in protein extracts of a stably transformed *M. polymorpha* line or to simultaneously detect MpSPI PBW and MpLIP5 in protein extracts of transiently transformed *N. benthamiana* leaves by immunoblotting.

### MpSPI localizes to P-bodies

**Figure 6:**
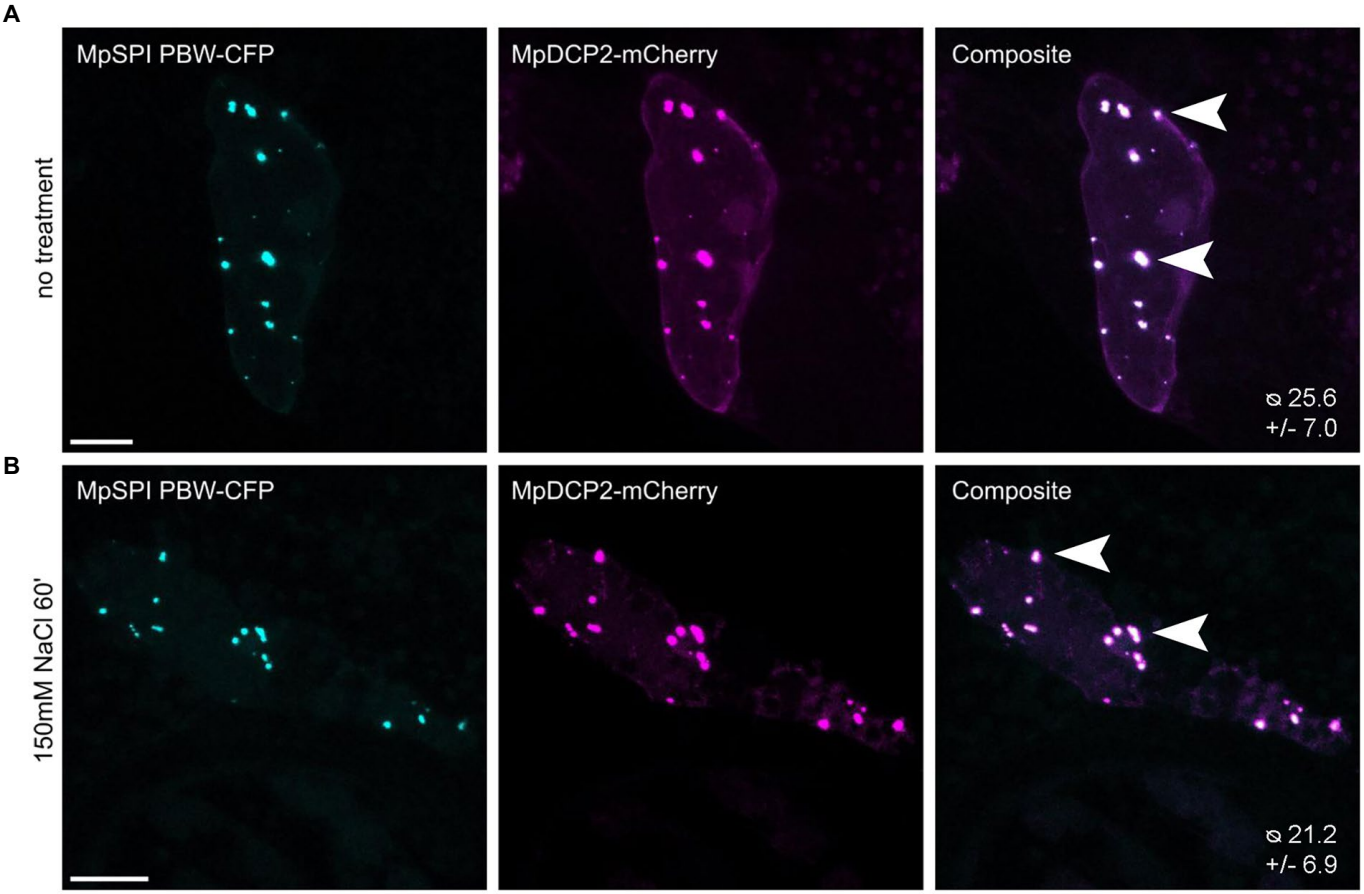
MpSPI PBW co-localizes with P-Bodies in *M. polymorpha.* Transient co-expression of MpSPI PBW with MpDCP2 in Tak-1 epidermal cells **(A)** under non-stress conditions and **(B)** salt stress (150 mM NaCl for 60 min). MpSPI PBW granule number in the co-bombardment shown in **A** and **B** was determined in *n* = 14 cells before and after salt treatment. No significant difference in granule number was detected with a two-tailed, unpaired Student’s *t*-test at *p* < 0.05. Arrowheads indicate areas of co-localization. Scale bars: 20 μm.

In *A. thaliana*, the PBW domain of SPI localizes to P-bodies under salt stress and differentially regulates the stability of RNAs in this context (Steffens et al., 2015). To test whether this is also the case in *M. polymorpha*, we transiently co-expressed MpDCP2 and MpSPI PBW. We found a strong co-localization in punctate structures under normal conditions (Figure 6A). However, the number of P-bodies labeled by MpSPI PBW was not altered by salt stress (150 mM NaCl for 60 min; Figures. 6A,B). Interestingly, the stress granule marker AtUBP1B, which localizes to dots upon stress in *A. thaliana* (Nguyen et al., 2016), is also present in granules already under non-stress conditions in *M. polymorpha* (Supplementary Figure S7).

To further support an association of MpSPI with P-bodies, we tested protein–protein interactions of MpSPI with MpDCP1. However, we found no interaction in pairwise yeast two-hybrid and BiFC assays in *M. polymorpha*.

### Differentially expressed genes (DEGs) overlap in Mp*spirrig* and *Atspi* mutants

**Figure 7:**
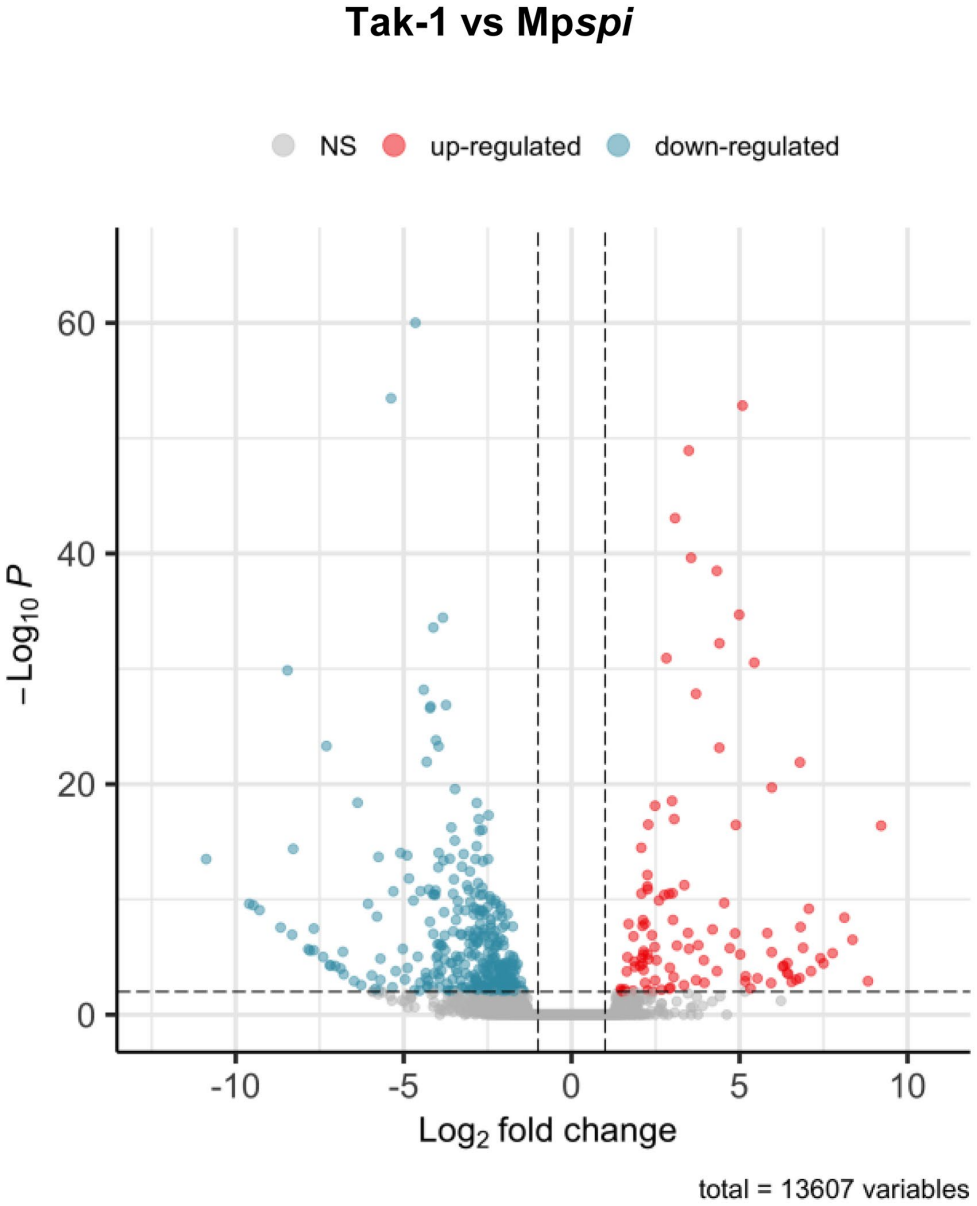
Volcano plot of differentially expressed genes between Tak-1 wild type and Mp*spirrig*. Data from three biological replicates per genotype. Of 20,574 expressed genes, 13,607 remained after quality filtering (> 10 reads across samples). Of these, 442 were differentially expressed at a threshold of 2-fold change (log2-fold change > 1; vertical dashed lines) at a significance level of 0.01 (horizontal dashed line). Of these, 104 showed higher expression (red) and 338 lower expression in Mp*spirrig* than in Tak-1.

The finding that MpSPI is relevant for morphogenesis and salt stress, similar to *Arabidopsis thaliana* and *Arabis alpina*, raised the question of whether SPI regulates common downstream genes. Previous experiments have shown that many genes are differentially expressed when comparing wild type and *spi* mutants in *A. thaliana* (Steffens et al., 2015). To explore this, we employed a comparative, genome-wide transcriptome analysis of Mp*spi* and wild type plants. Total RNA of both plant lines was extracted from three biological replicates of 14-day-old plants grown under normal conditions and subjected to RNAseq. All six samples had over 30 million reads each, with at least 19.5 million reads mapping to exonic regions. All samples clustered according to the genotype based on read counts (Supplementary Figure S8). Of the 20,574 expressed genes, 13,607 genes were retained after filtering (minimum ten reads in at least three samples; Figure 7). We identified 442 significantly DEGs (*p* < 0.01), comprising 104 upregulated and 338 downregulated genes, with at least 2-fold expression change between wild type and mutant (Supplementary Figure S9, Figure 7 and Supplementary Table S2). Consistent with our RT-qPCR analysis, the read count of Mp*SPI* was not significantly different in Mp*spirrig* samples and the wild type (Supplementary Figure S10).

**Figure 8:**
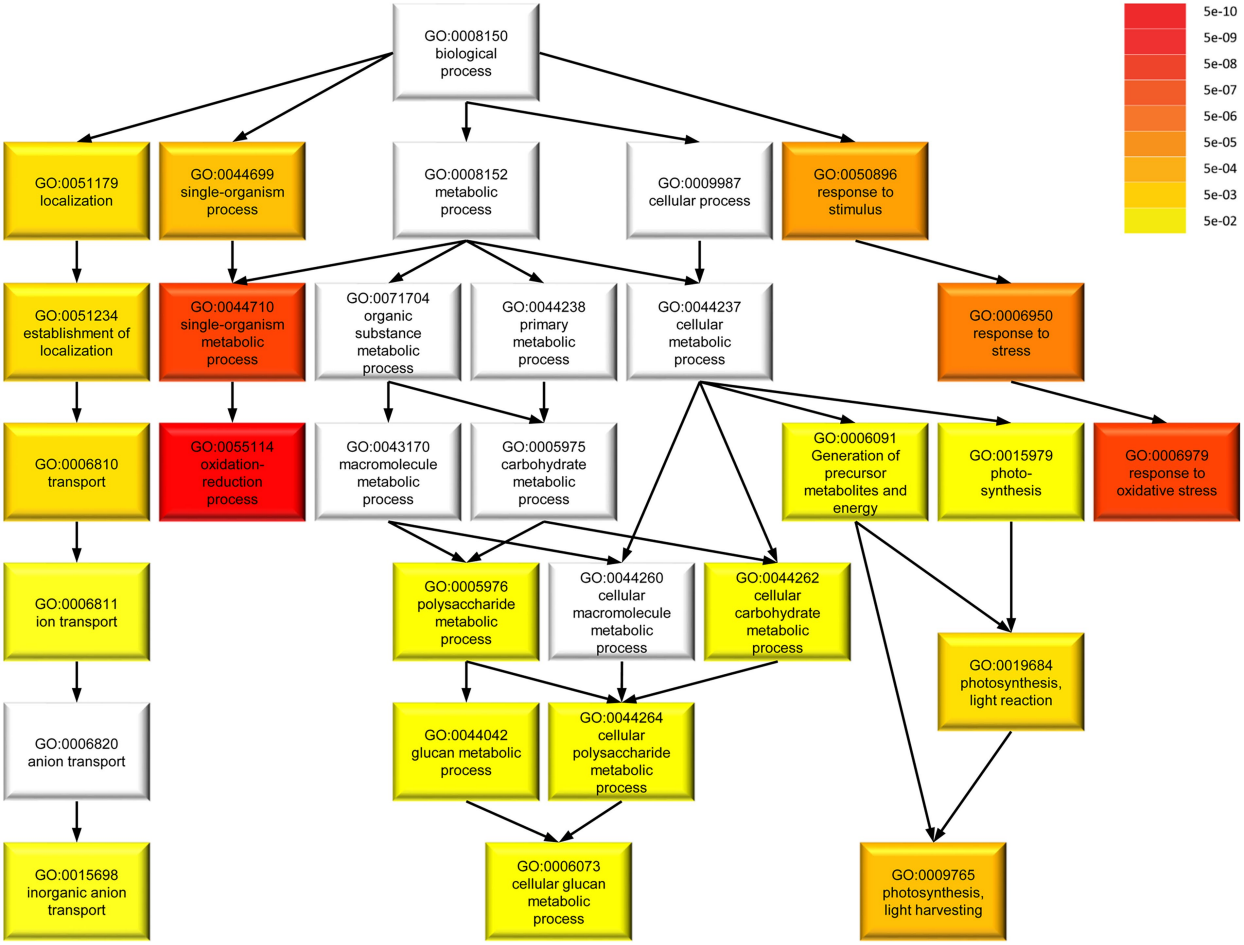
Gene ontology (GO) term enrichment of 217 DEGs in Mp*spirrig* with annotated terms in *M. polymorpha.* Darker colors in GO term categories represent higher enrichment indicated by lower false discovery rate (FDR). The analysis was performed with agriGO v2.0 on the background of the *M. polymorpha* locus ID v3.1 (Phytozome v11.0).

To determine common involvement in biological processes among the DEGs, we analyzed enriched GO terms (Figure 8). Among 217 *M. polymorpha* genes with annotated terms, we found a total of 20 significantly enriched GO terms in the category *biological processes* (FDR < 0.05, Figure 8). Interestingly, the six most strongly enriched GO terms oxidation–reduction process, single-organism metabolic process, response to oxidative stress, response to stress, response to stimulus, single-organism process, in that order (FDR < 5*E*–05, Supplementary Table S3) are present among the most strongly enriched GO terms of significant DEGs in *A. thaliana spirrig* mutants as well (FDR < 5*E*–05, Supplementary Table S4, derived from RNAseq data of Steffens et al., 2017). Next, we compared the set of significant DEGs (fold change > 2) in Mp*spirrig* to all significant DEGs in *A. thaliana spirrig* mutants (RNAseq data of Steffens et al., 2017). For 127 of the 442 DEGs, we could identify an *A. thaliana* ortholog using PhytoMine. Finally, we found a highly significant overlap (*p* = 4.08*e*–10) of 20 DEGs between Mp*spirrig and Atspirrig* (Supplementary Table S5).

## Discussion

In this work we studied the molecular and biological function of Mp*SPI* in the liverwort *Marchantia polymorpha* to enable an evolutionary comparison with two other plant species in which the homolog gene was characterized, *Arabidopsis thaliana* and *Arabis alpina.*

### Is Mp*SPI* a homolog of the *Arabidopsis SPI* gene?

We consider Mp*SPI* as an *AtSPI* homolog based on the finding that it clusters in the same group in a phylogenetic tree considering the BEACH domain (Supplementary Figure S1). MpSPI differs, however, from BDCPs in angiosperms at the structural level as it exhibits a FYVE domain in the C-terminus. FYVE domains target membranes by binding to phosphatidylinositol 3 phosphate (PtdIns(3)P; Gaullier et al., 1998). Both the human BDCP HsWDFY3/autophagy-linked FYVE protein (ALFY) and its Drosophila ortholog Blue cheese (Bchs) carry a FYVE domain downstream of the PBW domain and are involved in the clearance of aggregated proteins for autophagic degradation. In *A. thaliana*, none of the BDCP family members exhibit a FYVE domain (Teh et al., 2015). Recently, Agudelo-Romero et al. (2020) argued that the FYVE domain has been lost during land plant evolution because it has lost its essential function. This is consistent with our finding that protein fragments with and without the FYVE domain showed the same localization behavior. However, a more detailed molecular analysis would be necessary to explore this question.

### The dual role of *SPI* in morphogenesis and salt resistance Is evolutionary conserved

The Arabidopsis *SPI* gene has been described to be important in two processes that appear to be unrelated: cell morphogenesis and salt resistance (Steffens et al., 2015). The finding that a second, evolutionary distant Brassicaceae species, *Arabis alpina,* shows similar phenotypes in both processes suggested that the dual function is evolutionary conserved within that family (Stephan et al., 2021). The data reported in this study suggest that also Mp*SPI* in *Marchantia polymorpha* is involved in both processes. We found a clear hypersensitive response to salt stress. The role of Mp*SPI* in cell morphogenesis is evident from the short root hair phenotype. While the latter was reported in two independent alleles, we could only analyze one allele with respect to its response to salt stress. Our attempts to generate a second allele by CRISPR or to rescue the mutant phenotype failed for technical reasons. Therefore, we cannot exclude that the salt stress phenotype is due to a background mutation.

### The association of SPI with endosomal structures and P-bodies is evolutionary conserved

The molecular analysis of SPI in *A. thaliana* has revealed the co-localization of SPI protein with endosomes and P-bodies (Steffens et al., 2015, 2017). In addition, physical interactions of SPI with canonical endosomal proteins such as LIP5 and SKD1 and the P-body protein DCP1 were found suggesting that SPI exerts a molecular function in the two compartments (Steffens et al., 2015, 2017). We found no convincing evidence supporting the direct interaction of MpSPI with the interactors found in Arabidopsis. However, co-localization experiments with MpSPI revealed clear association with MpSKD1 and MpLIP5. This co-localization appears to be specific to a subpopulation of endosomes as the endosomal marker MpARA6 and MpRAB5 revealed only partial co-localization. This selective binding to MpSKD1 and MpLIP5 labeled endosomes may reflect a functional link. This may occur through MpLIP5 for which we found an interaction in yeast two-hybrid assays. Similarly, the co-localization of MpSPI with the P-body marker MpDCP2 may reflect a functional association, though the molecular basis remains elusive without the identification of an interaction partner. It is interesting to note, that in contrast to Arabidopsis, the association of MpSPI to P-bodies is not stress-dependent. Given that also the stress granule marker is associated in granules under non-stress conditions, it is likely that P-bodies and stress granules are constitutively present.

### Mpspi regulates stress response genes and a common set of genes also regulated by *Arabidopsis* SPI

Our genome-wide transcriptome comparison of Mp*spi* and wild-type revealed 442 significant DEGs with at least 2-fold change in expression. Our analysis revealed several strongly enriched GO terms that all suggest a role in stress responses. Strikingly, the significantly enriched GO terms in Arabidopsis are very similar suggesting that the transcriptional changes in *spi* mutants affect the stress response machinery in both species. We extended this analysis aiming to identify specific genes that are differentially regulated by both species in a SPI-dependent manner. Out of the 442 DEGs in Marchantia we could identify 127 orthologs out of which 20 DEGs were shared by Marchantia and Arabidopsis. While these genes do not immediately suggest the regulation of specific biological pathways, it is remarkable that 20 orthologous genes are regulated by SPI despite the enormous evolutionary distance.

## Data availability statement

The data presented in the study are deposited in the European Nucleotide Archive repository, accession number PRJEB51622.

## Author contributions

EK, LS, and MH designed this study. EK, LS, MS, and MH wrote the manuscript. EK and LS performed the research. EK, LS, and MS analyzed the data. All authors contributed to the article and approved the submitted version.

## Funding

This work was supported by the Deutsche Forschungsgemeinschaft Grant HU 497/15–1 (MH).

## Conflict of interest

The authors declare that the research was conducted in the absence of any commercial or financial relationships that could be construed as a potential conflict of interest.

## Publisher’s note

All claims expressed in this article are solely those of the authors and do not necessarily represent those of their affiliated organizations, or those of the publisher, the editors and the reviewers. Any product that may be evaluated in this article, or claim that may be made by its manufacturer, is not guaranteed or endorsed by the publisher.

## Footnotes

^1^ https://phytozome-next.jgi.doe.gov/phytomine
