# Supplementary material for: Functional analysis of the BEige and Chediak-Higashi domain gene MpSPIRRIG in Marchantia polymorpha
Source: Front Plant Sci. 2022 Sep 23;13:915268. doi: 10.3389/fpls.2022.915268 (PMC9537460; doi:10.3389/fpls.2022.915268)
Supplement: Supplementary file 1 [file Data_Sheet_1.PDF]

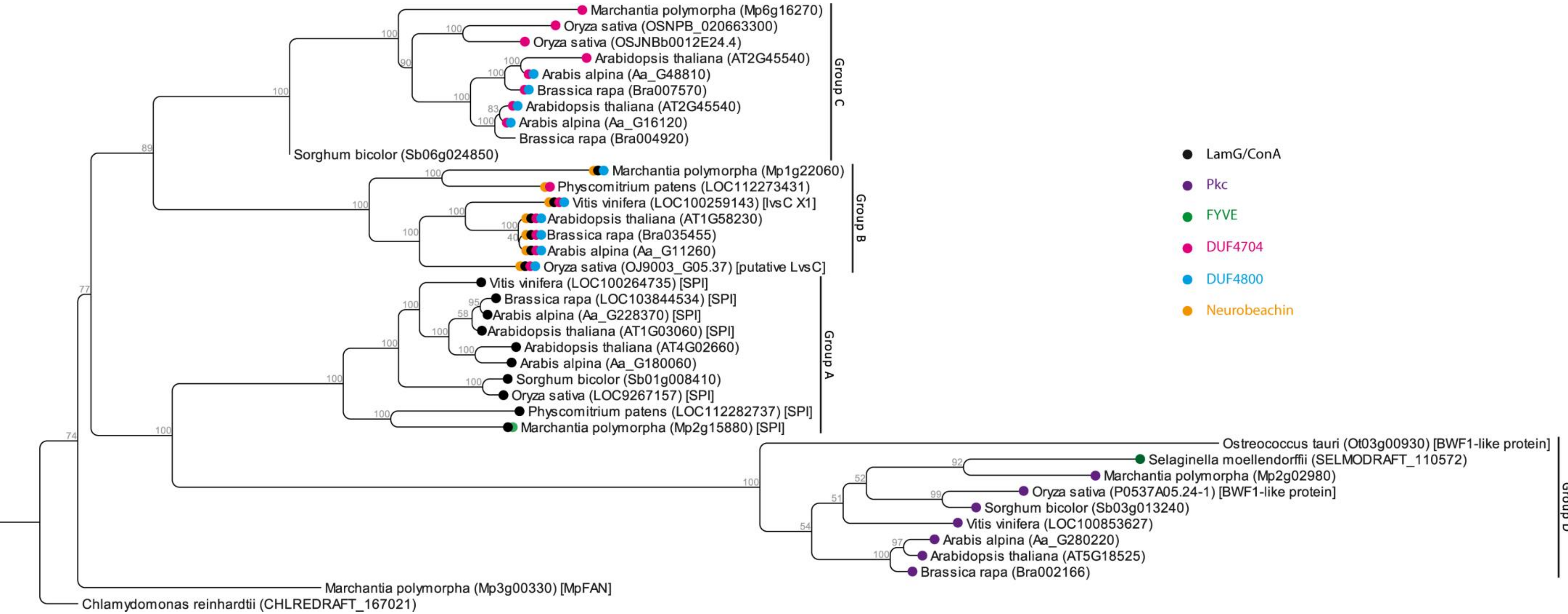

Figure S1. BEACH domain protein phylogeny in the plant kingdom. The phylogenetic tree was created with the Neighbor Joining algorithm using CLC Main Workbench 22. Bootstrap values are shown at nodes. Conserved domains are indicated by a colored dot. LamG/ConA - Laminin G/Concanavalin A superfamily domain, PKc – protein kinase catalytic domain, FYVE – FYVE domain, DUF – domain of unknown function. All depicted proteins contain the name-giving BEACH domain, often combined with a Pleckstrin Homology domain (groups A-C), WD40 repeats and Armadillo repeats (see also Figure 1).

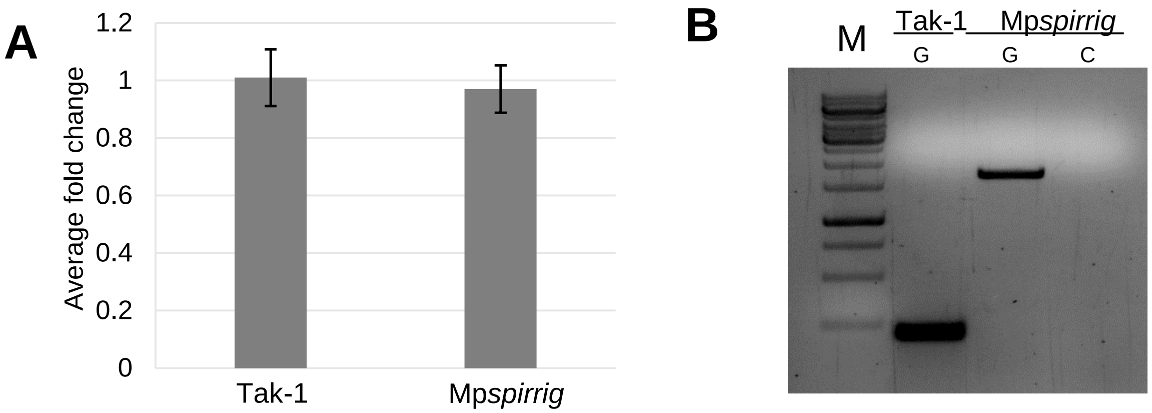

Figure S2. Mp*SPI* transcript analysis. **(A)** Mp*SPI* expression levels were determined in whole wild-type and mutant thalli by qPCR using a primer pair located downstream of the T-DNA insertion site in Mp*spi* (see Fig. 1B for primer location). Normalization was performed against Mp*APT3* and Mp*ACT7* (Saint-Marcoux et al., 2015). Transcript levels were not significantly different (Mann-Whitney-U test, $p<0.10$). **(B)** Qualitative RT-PCR with a primer pair spanning the insertion site in Mp*spi* (see Fig. 1B for primer location). The PCR was performed on genomic DNA (G) of Tak-1 and Mp*spirrig*. No PCR amplification was found with Mp*spirrig* cDNA (C). The integrity of the cDNA was confirmed in the qPCR experiments (A) for which the exact same RNA samples were used at the same time.

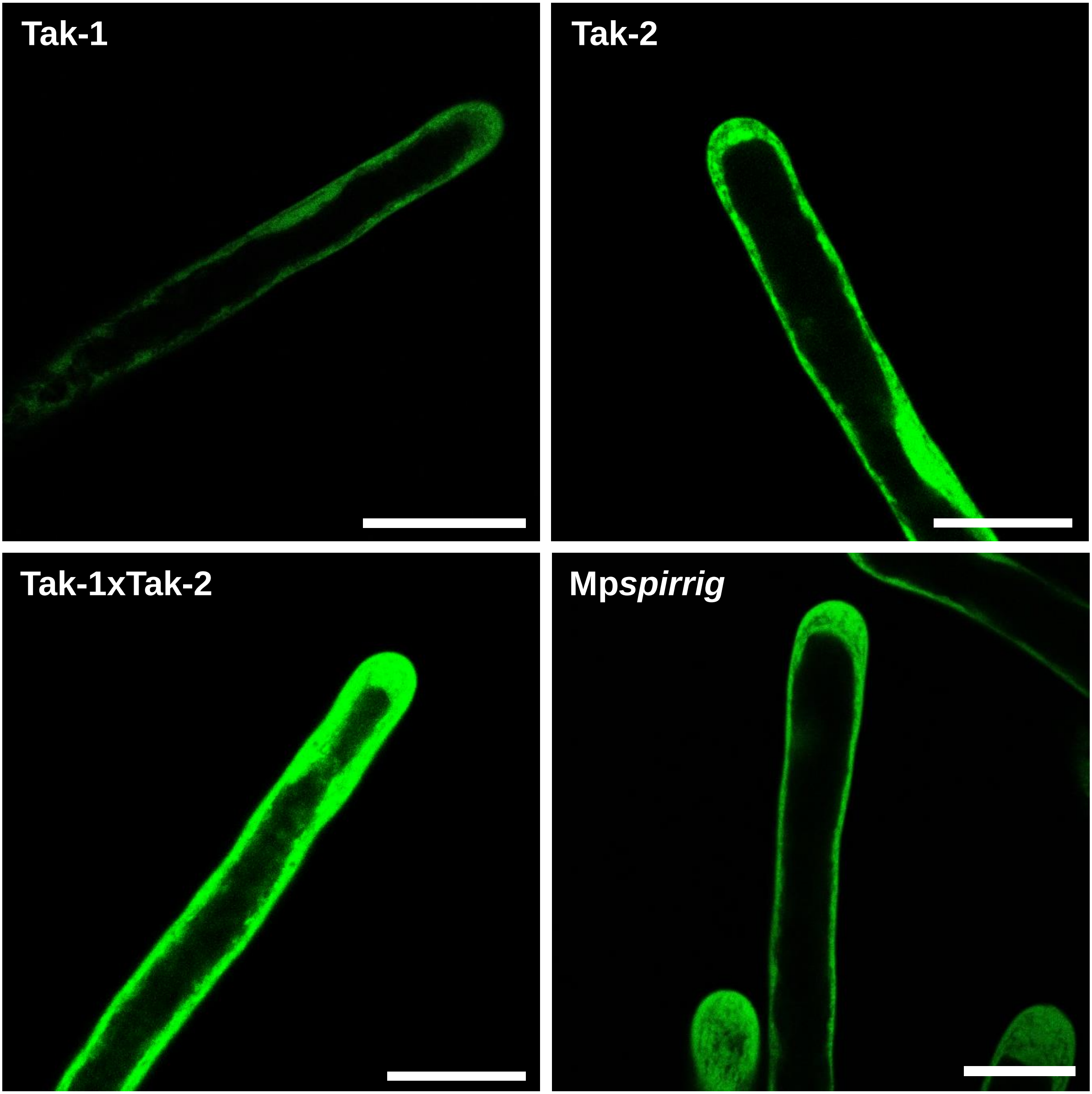

Figure S3. Vacuoles in rhizoids of Mp*spirrig* are not fragmented. Confocal images of fluorescein diacetate (FDA)-stained, 5-day-old gemmae. FDA stains the cytoplasm and vacuoles appear black. Vacuoles in rhizoids are intact and not fragmented in Mp*spirrig,* Tak-1, Tak-2, and F1 crossings of Tak1xTak-2. Scale bars: 25 µm.

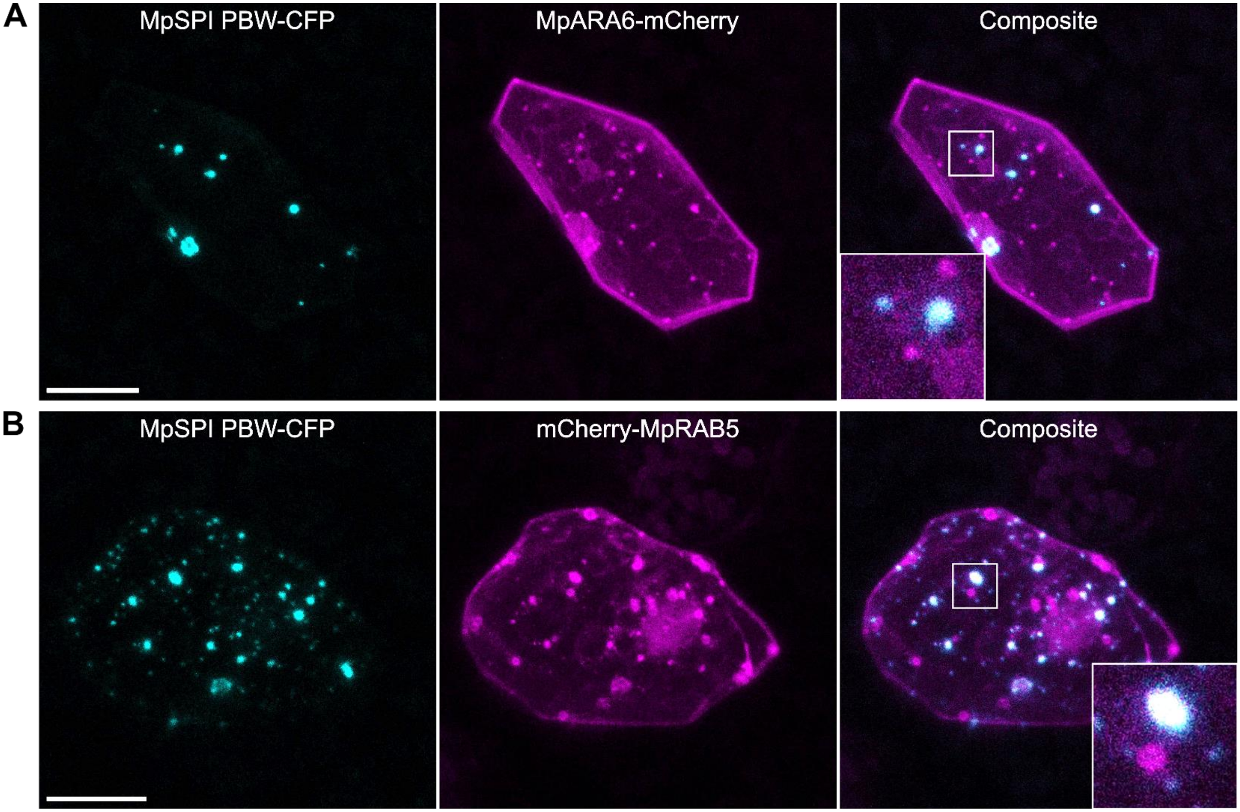

Figure S4. MpSPI PBW partially co-localizes with endosomes in *M. polymorpha.* **(A)** Transiently expressed, CFP tagged MpSPI PBW partially co-localizes to endosomes labeled with mCherry tagged MpARA6 in Tak-1 epidermal cells under normal conditions. The insert shows a higher magnification of the box to highlight non-overlapping fluorescent signals next to co-localization of both proteins. **(B)** Transiently expressed, CFP tagged MpSPI PBW partially co-localizes to endosomes labeled with mCherry tagged MpRAB5. The insert shows a higher magnification of the box to highlight non-overlapping fluorescent signals next to co-localization of both proteins. Scale bars: 20 µm.

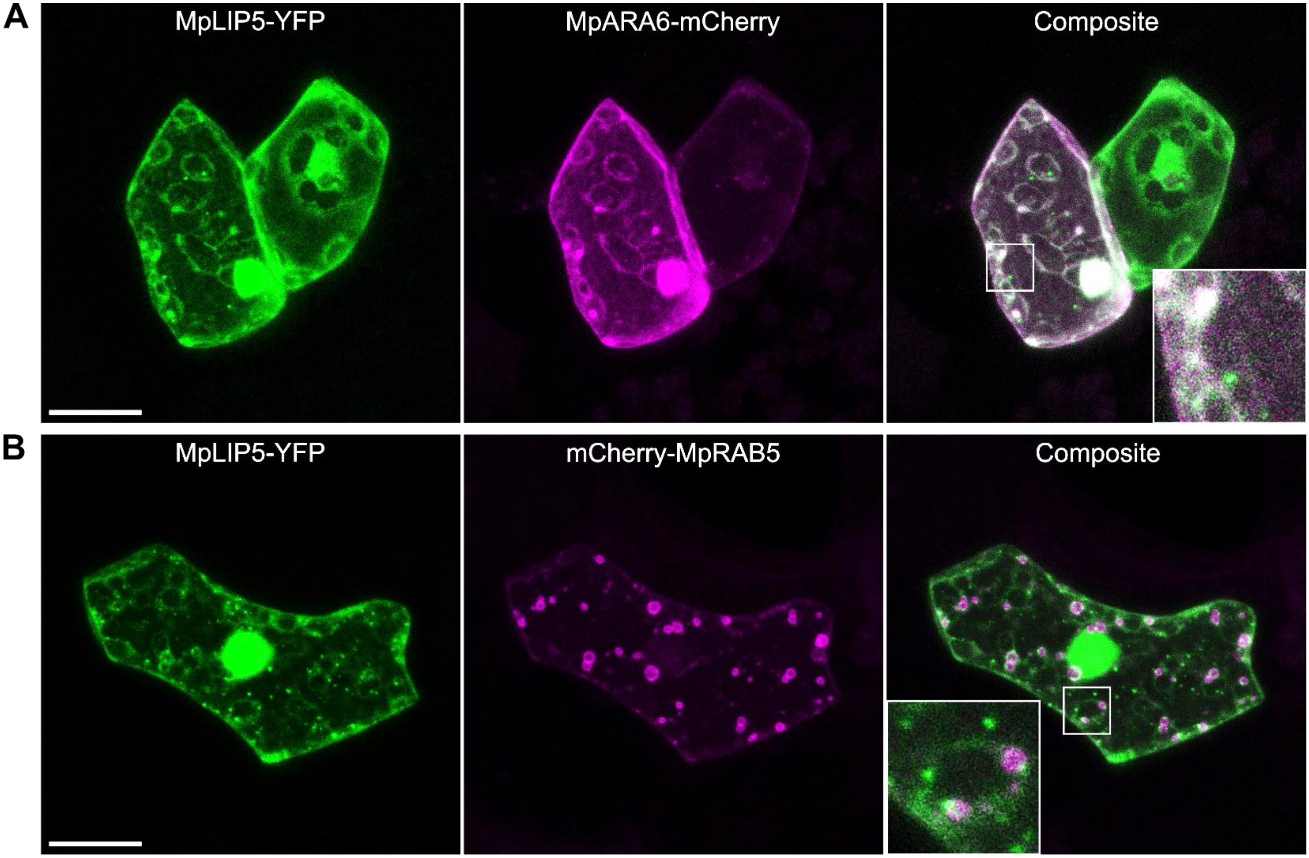

Figure S5. MpLIP5 partially co-localizes with endosomes in *M. polymorpha.* **(A)** Transiently expressed, YFP tagged MpLIP5 partially co-localizes with endosomes labeled with mCherry tagged MpARA6 in Tak-1 epidermal cells under normal conditions. The insert shows a higher magnification of the box to highlight non-overlapping fluorescent signals next to co-localization of both proteins. **(B)** Transiently expressed, YFP tagged MpLIP5 partially co-localizes with endosomes labeled with mCherry tagged MpRAB5 in Tak-1 epidermal cells under normal conditions. The insert shows a higher magnification of the box to highlight non-overlapping fluorescent signals next to co-localization of both proteins. Scale bar = 20 µm.

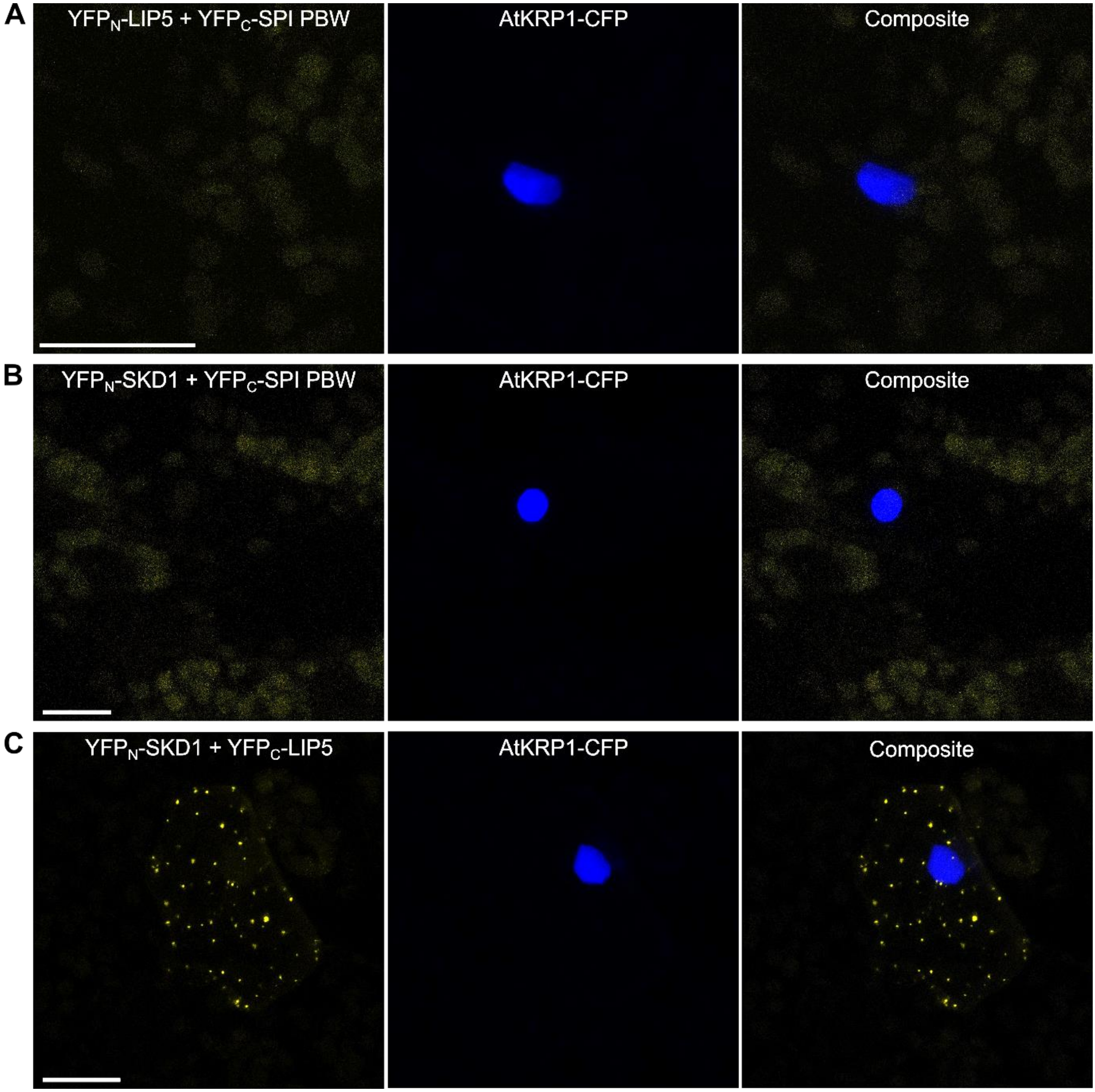

Figure S6. BiFC interactions between MpSPI PBW and ESCRT components were not detectable in *M. polymorpha*. Transient co-transformation of BiFC fusion constructs of **(A)** MpLIP5 and MpSPI PBW and **(B)** MpSKD1 and MpSPI PBW showed no fluorescent signal in epidermal Tak-1 cells. **(C)** Transient co-transformation of BiFC fusion constructs of MpSKD1 and MpLIP5 revealed an interaction in punctate structures in epidermal Tak-1 cells. To monitor successful transformation of cells the AtKRP-CFP was included. Scale bars: 20 µm

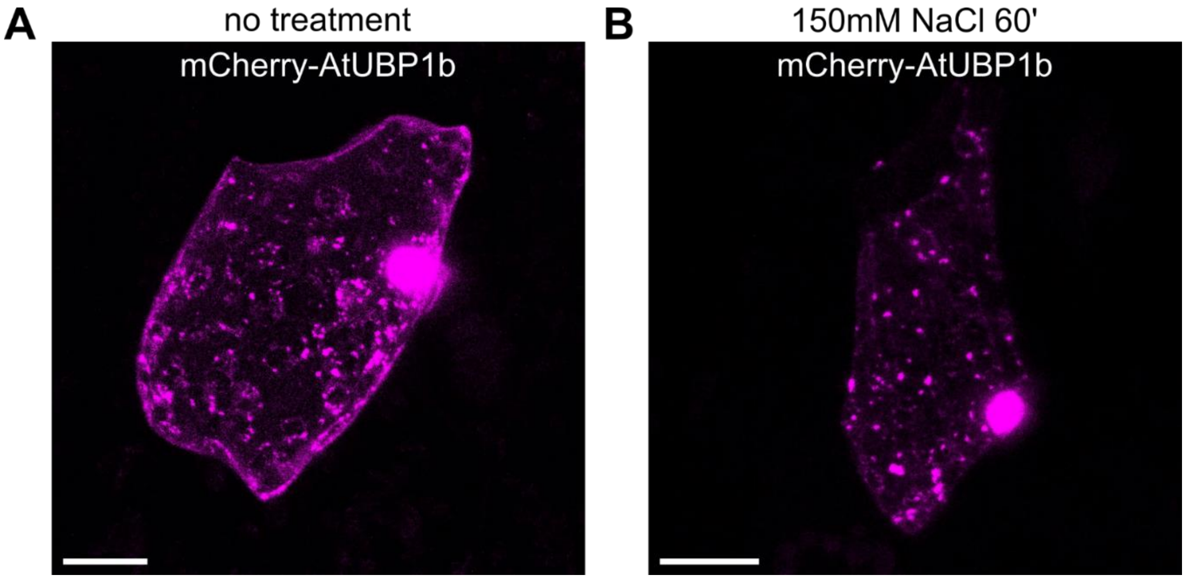

Figure S7. The stress granule marker AtUBP1b localizes to granules under normal conditions in *M. polymorpha*. Transiently expressed, mCherry tagged AtUBP1b localizes to dot-like structures under **(A)** normal conditions and **(B)** salt stress (150 mM NaCl for 60 min) in Tak-1 epidermal cells. Scale bars: 20 µm.

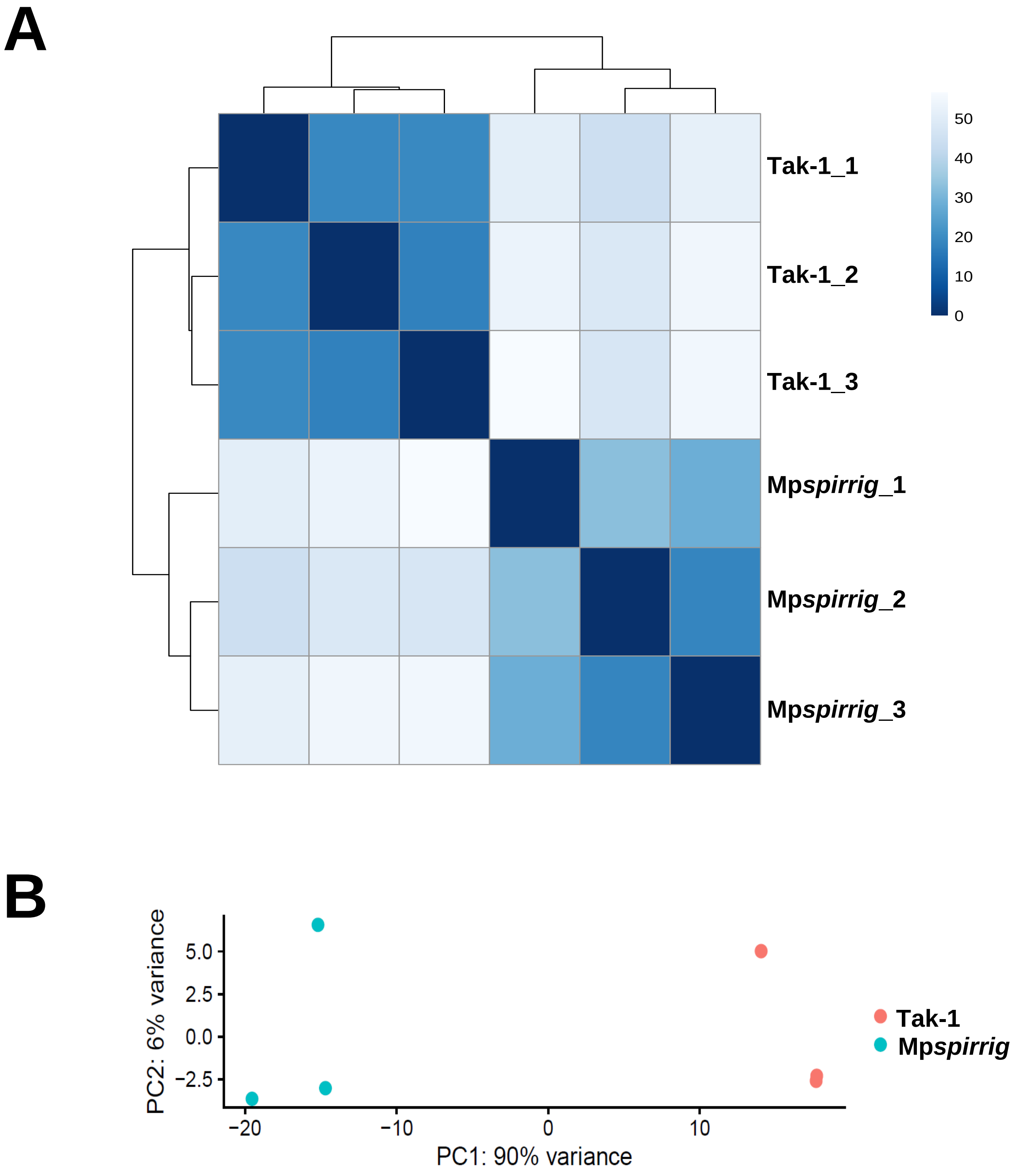

Figure S8. RNAseq data clustering of all replicates. **(A)** Heatmap of sample similarity based on Euclidean distance between samples. **(B)** Principle component analysis (PCA) on read counts presenting Tak-1 wild-type replicates with red dots, and Mp*spirrig* mutant replicates with blue dots. The 1st PC explains 90%, the 2nd PC 6%.

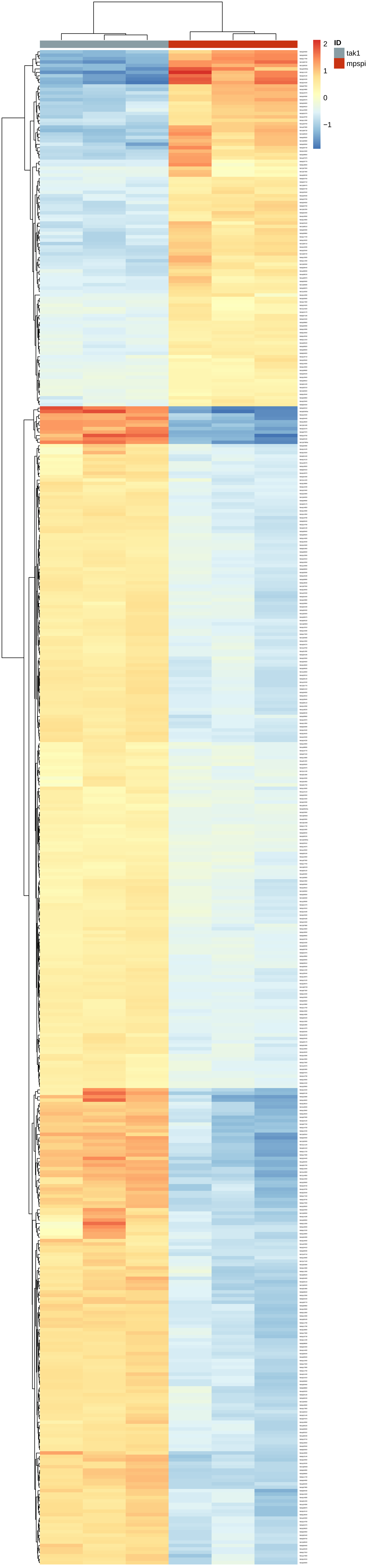

ID
tak1
mpspi
2
1
0
−1

Figure S9. Heat map of 442 genes with at least 2-fold change in expression between Tak-1 and Mp*spirrig.* The map shows the deviation of the average of each row.

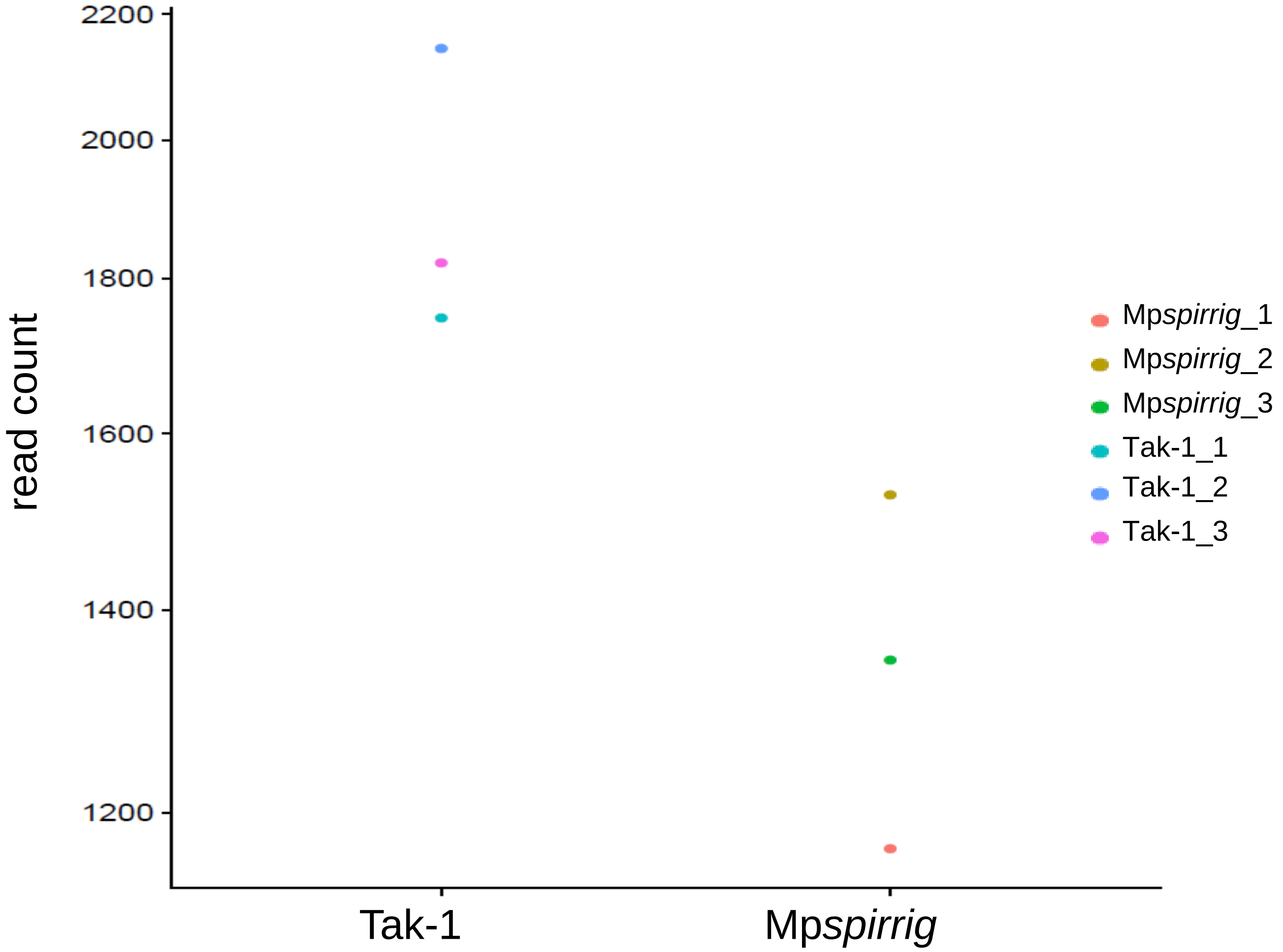

Figure S10. The read count of the Mp*SPIRRIG* transcript. Read count was determined in Tak-1 and Mp*spirrig* replicates in the RNAseq and does not significantly differ between the genotypes.
